# Meningitis

**Published:** 1906-06

**Authors:** James B. Baird

**Affiliations:** Atlanta, Ga.; Professor of the Principles and Practice of Medicine in the Atlanta Colleg Physicians and Surgeons


ATLANTA

### Journal-Record of Medicine

Successor to Atlanta Medical and Surgical Journal, Established 1855, and Southern Medical Record, Established 1870.

OWNED BY THE ATLANTA MEDICAL JOURNAL CO.

Published Monthly.

VOL. VIII. JUNE, 1906. No. 3.

BERNARD WOLFF, M.D., EDITOR, Nos. 819-20 Prudential.

M. B. HUTCHINS, M.D., BUSINESS MANAGER, 1007-1008 Century Bldg.

E. G. BALLENGER, M.D., ASSOCIATE EDITOR AND ASSISTANT MANAGER.

### ORIGINAL COMMUNICATIONS.

### MENINGITIS.*

BY JAMES B. BAIRD, M.D., ATLANTA, GA.

Professor of the Principles and Practice of Medicine in the Atlanta Colleg[illegible] Physicians and Surgeons.

The term meningitis signifies inflammation of the meninges of the brain, of the spinal cord, or of both, and it should be qualified by the prefixes cerebral, spinal or cerebro-spinal, as indicative of its location.

The clinical forms usually recognized are the traumatic, secondary, tubercular, simple and epidemic.

It is understood, however, that attention will be directed this evening especially to a consideration of epidemic cerebro-spinal meningitis or cerebro-spinal fever, and that for the purposes of the present discussion my remarks will be addressed particularly to this manifestation of the affection, referring only incidentally to other forms.

*Read before the Fulton County Medical Society.

Cerebro-spinal fever, sometimes designated spotted fever or petechial fever from the occasional presence of minute cutaneous hemorrhagic extravasations, is a severe infectious disease which has been recognized and studied for the last one hundred years, being first systematically described in 1805 by Vieusseaux, of Switzerland. Prior to that date the disease was confounded with typhus fever, although about the middle of the eighteenth century, under the name of hospital or jail fever, an epidemic which was probably an outbreak of cerebro-spinal fever, was described by a writer of that time.

More than forty years ago the late Dr. Austin Flint said of the causation: "The prevalence of the disease as an epidemic implies the existence of a special cause. With respect to the source of the special cause and the circumstances concerned in its production, we have no positive knowledge." In a subsequent edition of his classical work, some fifteen years later, with prophetic vision, characteristic of the wise and scholarly author, he elaborated his views on this point in the following paragraph: "All at present known of the pathological character of epidemic cerebro-spinal meningitis is embraced in the statement that it is an infectious disease, and belongs to the essential fevers. The inflammation of the piamater of the brain and cord is a local manifestation or an effect of an underlying, general morbid condition. It is reasonable to suppose that the specific infectious agent is a micro-organism, but with the exception of a few observations of microccoci in the exudation, nothing is positively known concerning such an organism. It must be admitted that the phenomena of the disease are largely symptomatic of the cerebro-spinal inflammation. Nearly all who have observed the disease concur in the belief that the causation does not involve a contagium.

"Facts pertaining to the localization of epidemics, and the occasional occurrence of sporadic cases, show conclusively that auxiliary causes which pertain to overcrowding, defective ventilation of dwellings, poisoning of the atmosphere by sewer emanations, etc., have a potent causative influence in association with the special cause, and it is an important fact that these auxiliary causes are by no means always confined to the habitations of the poor."

It seems now conclusively established that the specific cause of cerebro-spinal fever is the diplococcus intra-cellularis meningitidis, sometimes called meningo coccus. This organism can be isolated, according to various observers, from the spinal fluid, the meninges of the brain and spinal cord, the blood, the joint lesions and the nasal mucus.

It must be admitted, however, that in certain sporadic cases of meningeal infection presenting all the symptoms of the epidemic disease and clinically indistinguishable from it, only the strepto-coccus, the staphylococcus, the pneumo-coccus, the bacillus of influenza or the typhoid bacillus, and possibly others, have been found to explain the attack, and all of these organisms appear to hold etiological relations to meningitis, hence if belief in the meningo coccus as the specific, essential and sole cause of cerebro-spinal fever is unconditionally accepted, it must, of necessity, be recognized as a fact that all cases of cerebro-spinal meningitis can not be regarded as cerebro-spinal fever, and the differentiation must rest upon microscopical proof, or upon the existence of a prevailing epidemic, which may be received as fair presumptive evidence of the nature of the infection.

Cases which run a rapid course and reach an early fatal termination, present no appreciable anatomical lesion beyond an intense hyperæmia of the membranes of the brain and cord. In all, save in abortive or rapidly fatal cases, a transudation of serum containing more or less fibrin and pus occupies the sub-arachnoid space and the cerebral ventricles. As in inflammatory products of this kind elsewhere, either one or the other of these constituent parts may predominate, giving rise to a product chiefly serous, chiefly fibrinous, or chiefly purulent. Usually the exudate is most abundant at the base of the brain and on the posterior surface of the cord, thickly coating the posterior nerve roots and, as a rule, it is deposited more liberally in the dorsal and lumbar than in the cervical regions. I have occasionally found in autopsies a considerable quantity of fibrino-purulent matter spread as a creamy layer over the entire convex surface of the cerebrum, and the cortical cerebral substance, as well as the dura matter, and sometimes involved in the morbid changes.

Cases of this disease differ widely in severity, and in the relative prominence of the associated symptoms. The period of incubation is not known. The invasion is usually sudden. The attack begins as a blow from some mechanical force, and is in full sway before it can be realized. Occasionally premonitory symptoms may be observed for a few hours or a few days. These are headache, more or less severe, and which may be chiefly frontal, chiefly occipital or involving the whole head, backache and anorexia. The initial symptoms are a chill, intense headache, vomiting, fever, rigidity of the post-cervical muscles, which, with subsequent tonic spasm of the spinal muscles, may cause complete opisthotones. Muscular tremor and twitching, and temporary strabismus, are not infrequent. Convulsions, sometimes often repeated and of great severity, may characterize the onset in children. General convulsions are rare in adults, though spasm of individual muscles, or special groups, may occur. Delirium from the very beginning is frequent. It may be furious—maniacal in severity, or be shown only by slight confusion of ideas or mild incoherence. Coincidentally with the accumulation of inflammatory products, the period of excitement is succeeded by a stage of dullness or stupor, more or less profound and ultimately, perhaps, by coma.

The temperature is variable. It may fluctuate, or be continuously either high or low. The pulse may be full and strong, or rapid and feeble. The quality is apt to change as the case progresses, frequently indicating asthenia. Herpes about the lips and face, or in other situations, is very common. Indeed, herpes facialis possesses diagnostic importance.

Petechiae, purpura and rose-spots pertain to the cutaneous history of the disease. The urine is often albuminous. Cerebro-spinal fever may attack any age, from infancy to the octogenarian, though it is more common in young subjects than in adults, or at either extreme of life. The preponderance of evidence is in favor of its greater prevalence in the winter and spring, as compared with other seasons. This is certainly true of this locality. Epidemics are restricted in extent. The disease does not follow lines of travel, and exists at times in country districts in preference to populous communities. It is not generally considered contagious, in the sense that it is communicated directly from one individual to another. I have never known of an instance where it could be so traced.

The duration is from a few hours only to a few days, or, occasionally, to several weeks, except in the chronic form, when it may continue for months. Relapses are improbable, though instances of recurrence in the same individual are recorded. Convalescence may be prompt, or slow, steady or interrupted, complete or complicated, and followed by sequels of greater or less importance. Among the most common complications are pneumonia, pleuritis, pericarditis, parotitis and arthritis. The most dreaded sequels are mental impairment, blindness or deafness, the result of destructive inflammation involving the structures of the eye or ear, and entailing serious temporary or permanent injury.

The diagnoses, except in abortive or very mild cases, is attended with no difficulty. Cerebro-spinal fever may be discriminated from traumatic and secondary meningitis, from the tubercular form, and from typhoid fever and pneumonia, by careful attention to the symptoms and signs pertaining to this disease, and to the several affections enumerated above. The access is usually abrupt. When prodromal symptoms are present, they point plainly to the seat of the attack. The intense headache, referred to the frontal or occipital region, or to the whole head, early vomiting, with fever, photophobia, delirium, muscular rigidity or tremor, and stiffness or retraction of the neck, present a clinical picture which is almost conclusive. If the microscope should reveal the specific organism in the spinal fluid or the nasal mucus, associated with the usual symptoms, the determination would, of course, be complete.

Kernig's (1) sign, while not peculiar to cerebro-spinal fever, it does belong exclusively to spinal meningitis, and is said to be present in every case of spinal meningitis at some time in its course, it therefore constitutes a valuable auxiliary indication. The Babinski (2) or extension toe reflex is considered corroborative evidence, but it is inconsistent and not peculiar to meningitis. It was observed only twice in twelve or fifteen cases recently under my care. Like Kernig's sign, it may be unilateral. I am unable to state the significance of this one-sided manifestation.

By a process of exclusion, the affections most liable to be confounded with cerebro-spinal fever may be eliminated. The absence of traumatism, or the history of any disease that may hold a causative relation to meningitis, will exclude the traumatic and the secondary forms of the disease, and similar reasoning will serve to distinguish from the simple or sporadic and the tubercular forms, as well as from other diseases to which it may bear some resemblance.

Forty-four cases of cerebro-spinal meningitis have been treated in the Grady Hospital since January 1, 1905. Twelve last year, and the remaining thirty-two this year. They were distributed by months as follows:

| | |
|---|---|
| January | 2 |
| February | 4 |
| March | 14 |
| April | 13 |
| May | 10 |
| August | 1 |

(1.) Kernig's sign—Inability to fully extend the leg when the thigh is flexed at a right angle to the trunk.

(2.) Babinski's sign—Extreme extension of the toes when the sole of the foot is genly stroked with the hand.

The ages ranged from two to sixty years.

Five were under ten years.

Fifteen were between ten and twenty years.

Fifteen were between twenty and thirty years.

The remainder were over thirty years.

There were three whites and forty-one negroes.

Of the total number five recovered. Two left voluntarily—improved. Thirty-three died. Four remain under treatment.

Twenty-five were treated in my service. Five recovered. Two left voluntarily—improved. Fourteen died. Four remain under treatment—all improved.

One of my cases developed, as a complication, acute lobar pneumonia. The lung disease progressed as usual, and terminated by crisis—but the patient subsequently died.

In outlining an appropriate treatment, contrary to generally promulgated views, I shall mention first blood-letting—preferably by venesection, and, as a second choice, by leeches or wet cups. In the absence of circulatory contraindications, when it can be done in the very first stage, it seems to me to be the most prompt, direct and rational remedy at our command. Apart from considerations of elimination, it lessens, as nothing else can, the invariable, urgent and dangerous condition of extreme cerebral and spinal hyperæmia and seems to check the formation of the damaging products of inflammation, and probably to promote their absorption.

The general rule applicable to the employment of bleeding is not violated. No fear of asthenia need be entertained, or, rather, under the circumstance, it may be absolutely disregarded. If it should fail to do good, it can do no harm. Cold to the head and spine should be continuously applied by means of ice bags, and I believe a blister to the back of the neck, using for this purpose the catharidal collodion, is serviceable and ought not to be omitted. Anodynes are usually necessary for the relief of pain. The hypodermic use of codeine or of morphine, or some other preparation of opium, by the mouth or rectum may be employed. Among the useful sedatives and hypnotics, the bromides and chloral stand first. Ergot and adrenaline have been advanced for the alleged effect of counteracting the diameter of the cranial and spinal blood vessels, and thereby bringing about a diminution of the congestion and controlling the consequent evils. Theoretically, they are appropriate. I have repeatedly used both remedies, but without clearly appreciable results.

In my experience the most trustworthy medication is found in mercury and iodide of potassium. I begin with these drugs early and push them to the point of tolerance. Stimulants and tonics may be called for and, with the addition of cod liver oil, this line of treatment should be followed during and after convalescence. Cathartics are, as a rule, demanded. They should be exhibited as required. The apartment occupied by the patient should be shaded, quiet and well ventilated. The regimen should be simple, digestible and sustaining.

I know of no one who advises lumbar puncture as a therapeutic resource. It is recommended for diagnostic purposes, but it is unnecessary on this ground. It is not an entirely innocent procedure, as is frequently asserted and parrot-like reiterated. Collapse and sudden death have followed its use, and a satisfactory and accurate diagnosis should and can be made from clinical data without multiplying the misfortunes or increasing the risks of the victim of this dread malady. Neither is it justified solely upon the score of its spectacular qualities. It has been proposed, after drawing off the spinal fluid, to wash out the cavity with a normal salt solution, and then to throw in an antiseptic. A weak solution of lysol has been used in this manner, but the evidence of benefit is not, by any means, conclusive.

The experimental use of dipththeria antitoxin has been tried, and beneficial results have been alleged to follow this mode of treatment. Its advantages have not been satisfactorily demonstrated, and it is probably of no value. Certainly, the claims of its advocates have not been established.

In cases of mixed or of streptococcic infection, the serum treatment may offer some hope, and may be, in some degree, helpful. Future observation will ultimately settle the question of its utility.

I believe that it is in this direction that we must look for improved therapeutics of this disease.

DISCUSSION OF DR. BAIRD'S PAPER.

*Dr. Block:* In the cases which I have seen in this epidemic there has been an entire absence of the skin lesions, except dermatagrophia and herpes. While most of the cases have been very severe, I have seen several very mild cases in which a recovery has taken place in from three days to a week. In two cases there existed local paralysis. In one case it began with a conjugate deviation of the eyes, followed by a complete left hemiplegia, and the case ended fatally (autopsy). In the other there was a left-sided facial paralysis, and hypoglossal paralysis, from which the patient completely recovered. (Dr. F. L. Campbell's case.)

I have seen one case of a relapsing character in which there have been three febrile periods. The patient has been ill now for about three months, and is apparently recovering.

Trifacial herpes has been a marked feature of two cases under my care. One of these died, the other is apparently recovering.

In regard to treatment, vasa constrictors, such as ergot, strychnia, or digitalis, have seemed either harmful or at least of no benefit.

Sporteine sulphate to support the heart has seemed beneficial. Ice-bags to the head, mercurial purges, and antipyrine, have all seemed beneficial.

In two cases in which I have used unguentum Crede on the head and spine, one recovered completely (the case of facial paralysis cited above), and the other is apparently recovering.

*Dr. Purse:* As with those preceding me, I thank Dr. Baird for his most able and instructive paper upon meningitis—singularly apropos during the epidemic now present.

I most heartily concur with Dr. Harris in believing that the source of invasion is through the nasal cavity, and that measures, to be preventive, must be directed to that place.

After studying the pathology of the disease, I also am of the opinion that any future method of success in the management of this dread malady must combine surgical and medical therapeutics.

*Dr. H. F. Harris:* As cerebro-spinal meningitis is a disease that is due to a specific micro-organism, it is obvious that it can not exist without the presence of this germ; the malady is, therefore, infectious, and must necessarily be to a certain extent contagious, nothwithstanding the fact that it is generally thought not to be so. The bacterium that causes it at best lives only a day or so outside of the human body, and it is therefore absolutely essential, in order for the disease to be propagated, that it be carried from one person to another.

The reason that the disease has not been thought to be contagious may be explained by the following facts: Recent investigation has shown that in many, if not all, cases of this disease the germ may be found in the nose and throat, where it sets up a condition resembling an ordinary bad cold; it is highly probable that the infection takes place in all instances in the nasal cavity first, and from this point of vantage ultimately makes its way into the skull. Now there is every reason to believe that in many, and probably in a great majority of cases, the germ goes no further than the mucous membrane of the nose, and the patient has as a consequence merely a bad cold. It is clear, however, that if another individual who was very susceptible to this organism should become infected from this person, he might go on and have the meningeal form of it. In other words, it is probably true that the vast majority of people who are attacked by this germ simply get colds as a consequence, and only now and then does a person get meningitis as a result; this explains why the disease has in the past appeared not to be contagious.

The facts just referred to are of much importance in combatting this disease. People who are exposed to those having meningitis should be exceedingly careful not to get on their persons any of the secretions that come from the patient, and during epidemics those who observe a bad cold coming on should give it prompt attention.

As regards treatment, it is perhaps not an overstatement to say that further than treating the patient symptomatically nothing can be done—there being, unfortunately, no drug that acts as a specific in this terrible disease. As the condition is practically one of abscess around the brain, the most rational treatment would appear to be the opening up of the skull and allowing free drainage, but whether practical experience will demonstrate that this is effective or not only time can show.

*Dr. W. S. Kendrick:* In pathological conditions dealing with an inflammatory action of serous membrane: Said inflammation is practically the same in its results as inflammations of serous membranes elsewhere, namely, pleural, peritoneum, etc. In acute pleuritis have dry form, or effusion of sero-lymph or purulent. As the result, have adhesions and frequently permanent thickening; same conditions obtain in meningitis, except meninges lie in immediate contact with brain and spinal cord. Great majority of these cases are purulent, pus following tract of nerves, blood vessels, and finding its way into the brain substance. Special nerves at base of brain often involved, and if patients recover such are thickened and compressed to such an extent that partial or complete blindness, deafness, etc., ensue. Many cases reach an early death, with but little injection of either brain or meninges. The reason is obvious. The patient, overwhelmed by general intoxication and great toxemia, succumbs within the first few hours or days, others linger on, growing better, but with new infections, frequently relapse and death finally ensues. The very few that recover, in my judgment, do not pass the point in the exudate of sero-lymph. It is partly or completely absorbed, and no chronic lesions left behind. Diagnosis is usually easily made. One peculiarity, temperature often irregular and reaches an unusually high point preceding death. Prognosis in typical cases nearly always unfavorable. The speaker does not believe that a single case which is purulent has any chance to recover, unless by means not now known. We are dealing with an abscess which covers the circumference of the brain, and frequently finds its way into both brain and cord substance. Drainage, if properly had, would offer the best result. Various sera or anti-toxins hold out but little hope, as the disease is frequently sudden and rapid in its ouslaught. From 15th of February to 1st of April (six weeks) fifteen cases admitted in my service at the Grady Hospital, two-thirds partly or completely comatosed when brought in, of whom fourteen died within a few hours to twenty-eight days. Lumbar puncture made on the most of them, without special reference to recovery. In the great bulk the fluid withdrawn was purulent. In some cases giving temporary relief from pressure and pain. As a diagnostic point it is of inestimable value, done with cocaine or chloroform subjects patient to no great inconvenience. The writer has no confidence scarcely in any line of general treatment, except that which relieves pain and puts the patient in the most comfortable condition. Mercury, potassic iodide, and similar remedies, have no more place than in a pleura or peritoneum, with acute or chronic thickening and adhesions.

*Dr. W. B. Armstrong:* I should like to ask Dr. Kendrick if the skull had been opened and drained in the cases of lumbar puncture, where considerable pus was found? And if not, would like to suggest that the Grady Hospital would offer a good opportunity for determining whether this procedure was of benefit or not.

*Dr. F. W. McRae*: The results of medical treatment of cerebrospinal meningitis are so unsatisfactory that my mind turns to surgical intervention as a possible source of help. I realize fully the difficulties in the way. I believe, however, good might come from drainage of the cranial and spinal subdural spaces, but not in the late cases such as are seen in the Grady Hospital. Surgical intervention to prove effectual must be resorted to early in the disease.

*Dr. R. R. Kime*: The death-rate in epidemics of meningitis depends, to a great extent, on the severity of the attack. I have seen severe cases die in a few hours with what is called "spotted fever." Again I have seen other cases get well. I remember one case especially that occurred several years since in which a boy of fourteen years had a severe attack, was in bed three months unconscious for six weeks of the time, temperature obstinately high, ranging at 104 to 105° for weeks, unless forced down by baths. He recovered and was in better condition after than before the attack. Other instances might be mentioned.

Cases and epidemics are more common in northern and western sections just after prolonged bad winters at the beginning of spring. The application of drainage as suggested in these cases to my mind meets with difficulties. If it succeeds at all it must be very early or very late in abscess formation. The character of the inflammation is such that the effusion and the adhesions that soon occur prevent drainage for any distance. The symptoms are not definite enough, as a rule, to locate the point or points for drainage. Beside, in opening the skull you only drain a very small area, as the different points of infection do not communicate.

As to bleeding, large doses of veratrium veride (Norwood's) and ergot given early with calomel and salines will secure equally if not more efficient results.

*Dr. J. C. Olmsted*: I have greatly enjoyed the scholarly paper of Dr. Baird, which so well sums up our present knowledge of this disease. While we seem to have discovered the germ of the disease, it is a melancholy reflection that as regards the treatment, we have not progressed beyond the measures resorted to some thirty or forty years ago. About every remedial agent mentioned by him was in use at that time. As regards surgical procedure, such as puncturing the spinal canal, and draining off that fluid, as approved by some, I must confess that the rationality of the procedure does not commend itself to me. It is doubtless practicable to drain off the cerebrospinal fluid by puncture, but I would remark that this procedure cannot drain off the dense plastic and purulent effusion, so often found coating the convexity of the brain, and infiltrating its sulci; infiltrating also the nerve fibres, which swell, with diminution of the white substance of schwann, and often destruction of the axis cylinders. Again, some cases are observed so suddenly and violently overwhelming, "fondrayant" as the French call them, as to indicate a most profound toxemia, and *not* mere pressure effects. Post-mortem examination frequently reveals in such cases merely intense engorgement of the membranes of the brain and spinal cord. In fact, cases are recorded, like that reported by L. Emmett Holt, in which the symptoms observed during life, by no means corresponded to what would have been effected, from the extensive lesions observed post-mortem. In view of the disturbance of the circulation and the intense engorgement of the blood-vessels of the membranes in the first stage of the disease, I believe that at this time, general blood-letting may be of great service. As regards remedies, I have none to suggest. The measures mentioned, free purgation by calomel, the use of chloral, the bromides and opium, are doubtless of use in meeting certain indications. I may add that I expect little from surgical methods in the treatment of this disease, but have hopes of the future in regard to antitoxic treatment along the lines of serum therapy.

*Dr. J. W. Duncan*: I have been much interested in the paper by Dr. Baird, and also in the discussion.

I have in time treated a number of cases of meningitis. Some have died and others have recovered. Having treated a case heroically all night and far into the next day, without apparent benefit (hoping against hope) and at last see a change for the better, and the case go on to complete recovery, has greatly encouraged me to do what I could in every case. I have had only one case during the present epidemic—a girl eleven years old. The attack began with convulsions. I did not see her until the second or third day. She was wildly delirious, opisthotonos well marked, temperature 102°. I gave her six drops Norwood's tincture veratrum hypodermically, and the 1/8 gr. morphine, which quieted her for eight or nine hours. The same was repeated two or three times at intervals of ten or twelve hours. I also had a cantharides plaster 2x6 inches applied to back of neck and down the spine, and let it blister well; used ice caps on head. I also gave calomel and soda freely the first day. Ergotole bromidia and inct. digitalis was given every three hours during the case. The opisthotonos disappeared after three or four days, after which the veratrum and morphia was omitted. She had a partial return to consciousness about the sixth day, and was able to recognize her father, mother and neighbors. She gradually lost strength and died about the twelfth day of the disease.

*Dr. E. C. Cartledge*: I will consume about two minutes of the time long drawn out in this discussion to offer nothing of aid toward curing this wretched meningitis. My patient was a healthy young man nineteen years old, whom it took three men to hold on the bed when I saw him at noon. He had been delirious since the previous midnight. He had worked the day before. Apomorphia, gr. 1-10 quieted him partially without nausea for about thirty minutes or more. M. XV. (or about thirty drops) of Norwood Tr. veratrum, hypodermically, quieted him for about an hour, during which time we succeeded in putting ten grains of calomel in his stomach. The veratrum brought his pulse from 140 to 80, sweated him freely, nauseated him a bit, and the congested cyanosed appearance became a more natural one. We were encouraged. The ambulance arrived to take him to hospital; about this time he became wild again. We now gave him hypo., morph. 1-4 and atropia 1-150 with apomorphia gr 1-10 and took him to the hospital. There we prized his clinched jaws apart and by means of the stomach tube we put into his stomach three to four ounces of salts, eight drops Fl. Ext. Gelsemium and two teaspoonsful of bromidia. Very partial and temporary control of his wild delirium was given by these heroic efforts, and he died in twelve hours after I saw him.

*Dr. L. C. Rouglin*: I desire to know if the writer of the paper observed any special feature of the countenance of the patients in this epidemic. During the epidemic of the disease in New York I saw most of the cases admitted at Bellevue, and those at Lebanon Hospital, and the expressionless face was a marked feature. Dr. Northrup calling it the "masque face" of meningitis. I suggest the use of callorgolum in the form of unguentum crëde by inunction. It has bactericidal action; it facilitates the interchanges of oxygen and hydrogen in the animal tissue fluids, effecting an energetic increase of oxidation in the organism, thereby depriving the ptomaines and similar bodies of their poisonous character, and it increases leukocytosis. It has been used with success in septicemia, puerperal fever and in other septic and infectious diseases.

Meningitis is strictly a septic, a toxic condition, and I believe that good results may be obtained by the use of this drug.

*Dr. C. W. Strickler*: There is one point concerning the treatment of meningitis that I don't think has received the attention it deserves, which is, the necessity of keeping these cases absolutely quiet, free from any excitement.

Dr. Westmoreland called my attention to this several years ago, and cited the following case: A patient of his who had had this disease and was convalescing, was unduly excited by a visit from several members of his family. Patient promptly relapsed and died.

I noticed this in two patients under my care, while house-surgeon at the Grady Hospital. A private case, who seemed about well, relapsed after reading a letter from home.

Convalescents, especially from this disease, should be kept absolutely quiet and free from any excitement whatever.

*Dr. Archibald Smith*: I agree with those who advocate early operation as the safest treatment for appendicitis. The first case I ever assisted in operating on served to impress this on me, and subsequent experience has strengthened the impression. The patient was a young man of good general health who had been in active work till noon of the day of his operation, when he consulted a man who happened to be attending surgeon to King's County Hospital. A diagnosis of appendicitis was made and he was sent to the hospital at once and operated on as soon as preparations could be made.

His appendix was completely gangrenous and ready to slough off, though he had only been troubled for a few days, and his symptoms had not been severe enough to make him stop work. He made a good recovery, but a few days, or even hours, would probably have put him beyond help.

*Dr. L. B. Clarke*: I am not certain that I understood Dr. Baird correctly in his desire to draw a distinction between the forms of acute meningitis, and would ask that he repeat that part of his paper.

I understand acute meningitis to be the same, whether it be the acute cerebrospinal type (the so-called epidemic form, known as cerebrospinal fever) or the primary sporadic or the secondary forms. These I understand to be but subdivisions of the one disease.

